# Unusual presentation of seminal vesicle abscess: a report of two cases

**DOI:** 10.11604/pamj.2023.44.14.37883

**Published:** 2023-01-06

**Authors:** Supradeep Narayanaswamy, Rajvi Ravi Goradia, Sujata Kiran Patwardhan

**Affiliations:** 1 Department of Urology, King Edward Memorial Hospital, Mumbai, India

**Keywords:** Seminal vesicle, abscess, groin swelling, perineal abscess, case report

## Abstract

Seminal vesicle abscesses (SVA) are a rare condition, and their diagnosis is challenging with non-specific clinical presentation. Only a few cases of SVA have been published. Here we report two cases of SVA. The first one is a 58-year-old male with HIV and diabetes who presented with painful swelling in the left groin for 15 days. The second patient was a 65-year-old man who presented with painful swelling in the perineum for 15 days. Both patients were radiologically (computed tomography scan) diagnosed to have SVA. The first one was treated via surgical drainage for groin abscess and SVA was treated conservatively with intravenous broad-spectrum antibiotics. The latter was treated with SVA transurethral drainage. The pus culture showed Escherichia coli. Postoperative antibiotic therapies were contented without complications. In conclusion, although SVA may be clinically unsuspected, cross-sectional radiologic imaging findings should not be underestimated in order to promptly initiate treatment.

## Introduction

Pathology of seminal vesicles is uncommon and often misdiagnosed [1]. Very few cases of seminal vesicle abscesses (SVA) have been reported in the literature [1,2]. Clinical manifestations of SVA are non-specific and patients usually present with fever, burning micturition, suprapubic or perineal discomfort, and a tender prostate on per-rectal examination [3]. Because of their vague clinical manifestations, many cases of SVAs are missed and their diagnosis requires a high index of suspicion [4]. SVA presenting as a groin swelling or a perineal abscess is rarely reported [5]. We report two such cases wherein SVAs were presenting as a painful swelling in the left groin in the first case and a perineal abscess in the second case.

## Patient and observation

### Clinical case 1

**Patient information:** a 58-year-old male presented with painful swelling in the left groin for 15 days. The swelling was insidious in onset and gradually progressed in size. He also complained of pus discharge per urethra and fever without chills. He was previously treated for lower urinary tract symptoms (LUTS) secondary to benign prostatic hyperplasia (BPH) and was on a combination of Tamsulosin and Dutasteride for 2 months. He was catheterized a month ago for acute urinary retention. He was diagnosed case of HIV (human immunodeficiency virus) and has been on antiretroviral therapy for the past 8 years. He is a diabetic with poor blood sugar control.

**Clinical findings:** the abdomen was soft, and there was no guarding or rigidity. Local examination revealed a warm, erythematous, tender swelling in the left groin extending into the left scrotum. The left testis and spermatic cord were felt separately and were tender to touch. He was circumcised and had a 16 Fr internal foley catheter per urethra. Rectal examination revealed Grade II prostatomegaly without tenderness, or suspicious nodules.

**Figure 1:**
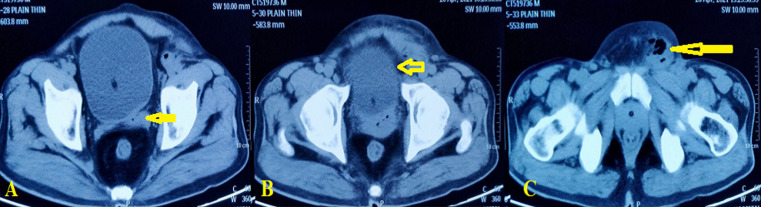
A) left seminal vesicle abscess (arrow); B) overlying bladder inflammation (arrow); C) groin abscess (arrow)

**Diagnostic assessment:** the complete blood workup revealed a hemoglobin of 6.9 g/dL, total leucocyte counts of 14900/microliter, and platelet count of 300,000 platelets per microliter. The serum creatinine was 6.5mg/dL and serum potassium was 6mEq/L at admission. Sonography of the inguinoscrotal region showed a heterogeneous area with air foci within the left inguinal region in favor of abscess formation. Contrast-enhanced computed tomographic (CECT) scan revealed a heterogeneous, hyperdense area of size 6.5x5x5 cm with air foci in the left groin. Another heterogeneous lesion of size 2X2 cm with air foci was seen in the left seminal vesicle and the left groin collection appeared to have tracked from this lesion (Figure 1).

**Therapeutic interventions:** firstly, patient underwent one session of hemodialysis. Then, a suprapubic catheter insertion and incision and drainage of the left groin abscess was performed. Intraoperatively we found pus in the left inguinal region tracking from the left seminal vesicle. He was started on intravenous Piperacillin (2000mg) and Tazobactam (250mg) every 12 hours along with intravenous metronidazole 500 mg thrice daily and was given intravenous paracetamol infusion for pain and fever control for 14 days.

**Follow-up and outcome:** the groin abscess was fully drained and the groin wound was allowed to heal secondarily with regular dressings. *Escherichia coli* and Pseudomonas aeruginosa were isolated from the urine culture while pus culture grew *Escherichia coli*. Both cultures were sensitive to piperacillin-sulbactam which was provided to the patient after adjusting the dose according to his renal function. The patient recovered completely and was discharged on the fourteenth postoperative day after clinical symptoms subsided and counts normalized. At discharge, he was started on a combination of tamsulosin and dutasteride one tablet at night, and was given oral ciprofloxacin 500 mg twice daily for 7 days. He was recommended to continue Antiretroviral therapy and was started on fixed-dose insulin for control of his diabetes. He followed up 1 month later, with uroflowmetry and transrectal ultrasonography to look for status of SVA. There was resolution of SVA. The uroflowmetry showed obstructed pattern with a failure of medical therapy. For that, he was then planned for transurethral resection of prostate (TURP) which was performed one month later. Following TURP, uroflowmetry showed improvement and the suprapubic catheter was removed one week later.

### Clinical case 2

**Patient information:** a 65-year-old male with complaints of painful swelling in the perineum for 15 days. He had LUTS secondary to BPH and was on treatment for the same. He is a diabetic and is on an oral hypoglycemic agent with negligent control.

**Clinical findings:** on examination, there was a tender, erythematous swelling in the right perineum. On rectal examination, the patient had grade II prostatomegaly with no tenderness or suspicious nodules.

**Figure 2:**
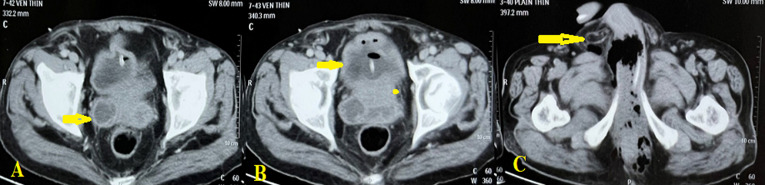
A) right seminal vesicle abscess (arrow); B) overlying bladder inflammation (arrow); C) perineal abscess (arrow)

**Diagnostic assessment:** the hemoglobin was 10.9 g/dL and leucocyte counts were 22,900/microliter. The blood urea nitrogen was 9.8 mg/dL and creatinine level was 1.1 mg/dL. Sonography of the perineum showed an abscess of size 4.5X3X3 cm. A CECT revealed an SVA of size 3X3 cm which had tracked into the perineum (Figure 2).

**Therapeutic interventions:** he was started on intravenous Ceftriaxone 1000 mg every 12 hours for 10 days along with intravenous amikacin 750 mg once daily for 5 days. He underwent incision and drainage of a perineal abscess via a cruciate incision over the perineum. He also underwent transurethral resection of the ejaculatory duct for SVA drainage. *Escherichia coli* was isolated from the pus which was sensitive to Amikacin.

**Follow-up and outcome:** his recovery in the postoperative period was satisfactory and he was discharged on the tenth postoperative day after clinical symptoms subsided and leucocyte count normalized. His diabetes mellitus was adequately controlled with regular insulin at discharge. He was asked to follow up in 1 month with uroflowmetry and transrectal ultrasonography. It showed no residual SVA. The uroflowmetry showed obstructed pattern, for that a combination of tamsulosin and dutasteride was started which caused significant improvement in his symptoms.

**Patient perspective:** the patients were happy with the successful outcome of the treatment.

**Informed consent:** written informed consent was obtained from the patients for participation in our study.

## Discussion

Seminal vesicle pathologic conditions, such as cysts, abscesses, and malignancy are infrequent [2]. SVA presents with non-specific clinical features like fever, dysuria, and perineal pain. Pandey *et al*. who did a literature review of 20 patients with SVA found 74% of patients having fever, 58% of patients having dysuria, and 32% of patients had tender prostate or epididymis-orchitis on clinical examination [6]. One case described by Saha *et al*. had a groin swelling like our own with pus tracking from left seminal vesicle [5]. But to our knowledge, no documented case exists where SVA presented as a perineal abscess. SVA presenting as a groin swelling or perineal abscess is rare and a high index of suspicion is needed for the diagnosis, particularly when they are prone to the development of an abscess. Their pathogenesis is not well known and is thought mostly to occur secondary to acute bacterial prostatitis or urinary tract infection [2].

Risk factors for SVA development include long-standing indwelling per-urethral catheter which may obstruct the ejaculatory ducts and allowing colonization by micro-organisms might promote urinary tract infection, immune-compromised status like diabetes, retroviral infection, use of steroids or immunosuppressant like in transplant recipients, malnutrition, and chronic alcoholism, and bladder outlet obstruction due to BPH or stricture urethra, recurrent and unresolved urinary tract infections, history of per urethral endourological intervention, and anatomical abnormalities particularly those of ejaculatory ducts [7,8]. Both our patients had LUTS secondary to BPH and the first one had immune-compromised status. The first patient was HIV positive, and had poorly controlled diabetes mellitus and renal failure with a high creatinine level; the second patient was an uncontrolled diabetic.

Without a specific clinical manifestation, diagnosis of SVA clinically is difficult and most often requires a high index of suspicion. *Escherichia coli* is the most common organism isolated in pus and urine cultures from these patients. Infection with Mycobacterium tuberculosis might cause SVA, particularly in endemic areas like India [9]. Digital rectal examination (DRE) in these patients usually reveals an enlarged prostate with tenderness over involved seminal vesicles. Diagnosis solely based on DRE is difficult. Radiological investigations like transrectal ultrasonography (TRUS), CECT, and Magnetic resonance imaging (MRI) will be required in most cases [2]. The lesions appear heterogeneous and hypoechoic with thick walls and septations in TRUS which can also guide therapeutic aspiration or drainage of pus [10]. The most common modality used for SVA diagnosis is CECT. SVA appears as a hypodense lesion with adjacent fat stranding with unilateral or bilateral seminal vesicle enlargement. MRI has higher spatial resolution and is superior when compared to TRUS or CECT for SVA diagnosing [11]. When either TRUS or CECT is inconclusive, MRI can be used for SVA diagnosis [12]. In our patients, clinical manifestations and DRE could not help us in SVA diagnosing, and ultimately, CECT was essential for the diagnosis.

Intravenous antibiotics with adequate gram-negative organism cover will suffice in most cases. If the patient doesn't respond to conservative management, drainage of the abscess is indicated. SVA can be drained per urethrally by endourological procedure like Transurethral resection of ejaculatory duct. [2]. Additionally, SVA can also be aspirated by TRUS or transrectal puncture [13]. The management should also focus on the improvement of immunity and address the predisposing factors the patients have. As in our case, both patients received consultation by an endocrinologist for poorly controlled diabetes, along with BPH therapy. These patients should be reviewed back for residual collection or abscess after completion of antibiotic course with TRUS. The predisposing factors such as immunosuppression, uncontrolled diabetes, HIV viral load, and indwelling catheters should also be followed up to prevent a recurrence.

## Conclusion

SVA is rare, and its diagnosis is difficult because of the non-specific complaints. SVA can present at unusual locations like a groin or perineal abscess, which need a high index of suspicion and should be included in the differential diagnosis in patients with risk factors like poorly controlled diabetes, indwelling catheters, and BPH. TRUS and CT scan are the imaging modalities of choice for diagnosis. Transurethral incision and drainage, percutaneous drainage, and TRUS-guided drainage modalities accompanied with broad-spectrum antibiotic of choice for treatment.
